# Impact of multiple cardiovascular medications on mortality after an incidence of ischemic stroke or transient ischemic attack

**DOI:** 10.1186/s12916-021-01900-1

**Published:** 2021-02-03

**Authors:** Tian-Tian Ma, Ian C. K. Wong, Cate Whittlesea, Kenneth K. C. Man, Wallis Lau, Zixuan Wang, Ruth Brauer, Thomas M. MacDonald, Isla S. Mackenzie, Li Wei

**Affiliations:** 1 Research Department of Practice and Policy, School of Pharmacy, University College London, London, UK; 2 Centre for Safe Medication Practice and Research, Department of Pharmacology and Pharmacy, Li Ka Shing Faculty of Medicine, The University of Hong Kong, Pok Fu Lam, Hong Kong; 3 Medicines Monitoring Unit (MEMO Research) and Hypertension Research Centre, University of Dundee, Dundee, UK

**Keywords:** Stroke, Combination drug therapy, Mortality, Cohort study

## Abstract

**Background:**

To manage the risk factors and to improve clinical outcomes, patients with stroke commonly receive multiple cardiovascular medications. However, there is a lack of evidence on the optimum combination of medication therapy in the primary care setting after ischemic stroke. Therefore, this study aimed to investigate the effect of multiple cardiovascular medications on long-term survival after an incident stroke event (ischemic stroke or transient ischemic attack (TIA)).

**Methods:**

This study consisted of 52,619 patients aged 45 and above with an incident stroke event between 2007 and 2016 in The Health Improvement Network database. We estimated the risk of all-cause mortality in patients with multiple cardiovascular medications versus monotherapy using a marginal structural model.

**Results:**

During an average follow-up of 3.6 years, there were 9230 deaths (7635 in multiple cardiovascular medication groups and 1595 in the monotherapy group). Compared with patients prescribed monotherapy only, the HRs of mortality were 0.82 (95% CI 0.75–0.89) for two medications, 0.65 (0.59–0.70) for three medications, 0.61 (0.56–0.67) for four medications, 0.60 (0.54–0.66) for five medications and 0.66 (0.59–0.74) for ≥ six medications. Patients with any four classes of antiplatelet agents (APAs), lipid-regulating medications (LRMs), angiotensin-converting enzyme inhibitors (ACEIs)/angiotensin receptor blockers (ARBs), beta-blockers, diuretics and calcium channel blockers (CCBs) had the lowest risk of mortality (HR 0.51, 95% CI 0.46–0.57) versus any one class. The combination containing APAs, LRMs, ACEIs/ARBs and CCBs was associated with a 61% (95% CI 53–68%) lower risk of mortality compared with APAs alone.

**Conclusion:**

Our results suggested that combination therapy of four or five cardiovascular medications may be optimal to improve long-term survival after incident ischemic stroke or TIA. APAs, LRMs, ACEIs/ARBs and CCBs were the optimal constituents of combination therapy in the present study.

**Supplementary Information:**

The online version contains supplementary material available at 10.1186/s12916-021-01900-1.

## Background

Stroke is the second most common cause of death worldwide and the third most common cause of death in the UK [1, 2]. According to Heart and Circulatory Disease Statistics 2020, over 1.3 million people in the UK have survived a stroke or transient ischemic attack (TIA) [2]. Optimal pharmacological therapy plays a key role in preventing the recurrence of stroke, cardiovascular events and reducing the risk of mortality. To manage the risk factors and to improve clinical outcomes, patients with stroke commonly receive multiple cardiovascular medications. Guidelines recommend antihypertensive, lipid modification and antiplatelet agents for the secondary prevention of stroke [3, 4]. The findings from the INTERSTROKE study identified hypertension as the most important risk factor for stroke with a population-attributable risk of 51.8% [5]. Evidence from a systematic review of randomised controlled trials (RCTs) suggested that antihypertensive treatment reduced recurrent vascular events by 21% in patients after stroke [6]. A large systematic review of observational studies and RCTs supported a short-term outcome benefit from statins [7]. Antiplatelet agents have been shown to prevent death and vascular events in patients with a high risk of cardiovascular disease [8], and dual antiplatelet therapy was suggested to be more effective on short-term outcomes than monotherapy in systematic reviews [9–11]. Although in routine practice, most patients are on combination therapy of multiple cardiovascular medications, the existing evidence from clinical trials has mostly focused on a single cardiovascular medication. The effect of combined antiplatelet agents and combined antihypertensive medications was only assessed in clinical trials for the prevention of stroke [12, 13].

A knowledge gap remains in identifying the optimal combination of medication therapy after ischemic stroke. It is unclear whether increasing the numbers or the classes of cardiovascular medications would have additional benefits on long-term survival. Further, the optimal constituents of combination therapy have not been comprehensively identified. This study aimed to investigate the effect of multiple cardiovascular medications on long-term survival after an initial ischemic stroke or TIA event.

## Methods

### Study design

A cohort study was conducted using The Health Improvement Network (THIN) database (now known as IQVIA Medical Research Data (IMRD)-UK database).

### Database

THIN is a primary care database that contains anonymised data from general practices across the UK. The database includes over 16 million patients from over 744 general practices. In 2013, the active patients in THIN represented approximately 6% of the UK population [14].

THIN includes information for each individual on demographics, diagnoses, prescriptions, referrals, laboratory tests, immunisations and the local area deprivation score (Townsend score) [15]. Primary care physicians and practice staff use a Read Code system to input and distinguish diagnoses, symptoms, investigations and lifestyle information in the electronic clinical notes. Prescription data are recorded via drug codes, and these can be identified by their generic name or by the British National Formulary (BNF) chapter [16]. THIN data have previously been used to study acute cardiovascular events [17].

### Study population

This study included patients with their first diagnosis of ischemic stroke or TIA between January 2007 and December 2016. Patients who were aged 45 or above and who had been registered for at least 3 years in the THIN database before the first stroke event were included in this study. We excluded patients who had a history of myocardial infarction (MI) before the first stroke or TIA event, who had died of all causes or who had an occurrence of a further cardiovascular event within the first 90 days after the first event of stroke or TIA. Follow-up of the included patients commenced at the date of the incident stroke/TIA event and ended until the earliest of 31 December 2016, date of registered death and the date of leaving the general practice during the study period. For each patient, the follow-up was divided into contiguous periods of 1 year, each defined with specific entry and exit points.

### Exposures and controls

Cardiovascular prescriptions were identified using drug codes in the THIN database. Each patient could contribute to several therapy categories, according to the cardiovascular medications issued at each entry point. Cardiovascular medications were identified based on all medications classified in the British National Formulary (BNF) Chapter 2 (cardiovascular system). Combination preparations were separated into their individual drug constituents.

We investigated the effect of combination therapy based on different numbers, classes and combination regimens on all-cause mortality. According to the numbers of cardiovascular medications (any medications identified based on BNF) prescribed in each 90-day exposure window, patients were stratified into groups of 0, 1, 2, 3, 4, 5 and ≥ 6 cardiovascular medications at each entry point. We then selected six evidence-based classes of cardiovascular medications commonly used for the secondary prevention of cardiovascular disease. The six classes of cardiovascular medications were antiplatelet agents (APAs), lipid-regulating medications (LRMs), angiotensin-converting enzyme inhibitors (ACEIs)/angiotensin receptor blockers (ARBs), beta-blockers (BBs), diuretics (DRs) and calcium channel blockers (CCBs) in stroke/TIA patients. Patients were stratified into groups of 0 (none of any cardiovascular medication) to 6 classes. Six classes of cardiovascular medications are APAs, LRMs, ACEIs/ARBs, CCBs, DRs and BBs exclusively. Patients who were on other class treatment were excluded from the study due to the complexity of the drug combination and few patients. Patients with one drug treatment or one class drug treatment were considered as the control group.

### Data extraction and confounders

Patient demographics, clinical characteristics within 1 year prior to each entry point and prescriptions within 3 months prior to each entry point were extracted from the THIN database. Confounding variables included age, gender, smoking status (never smoked, former smoker), alcohol consumption (never drank, current drinker, former drinker), body mass index (BMI) (mean, normal, overweight, obese and underweight), blood pressure (BP) status (normal; stage 1, 2 and 3 hypertension; and hypotension), total cholesterol (TC) status (optimal, intermediate and high), Townsend scores, history of hypertension, hyperlipidaemia, arrhythmia, heart failure, peripheral vascular disease, percutaneous transluminal coronary intervention, diabetes, dementia, chronic obstructive pulmonary disease, asthma, liver disease, peptic ulcer disease, rheumatoid arthritis and chronic kidney disease. Previous use of cardiovascular medications and nonsteroidal anti-inflammatory medications (NSAIDs) were also included.

### Statistical analysis

Data are summarised as mean (SD) for continuous variables and as frequencies (%) for categorical variables. Comparisons were performed using analysis of variance (ANOVA) for continuous variables and the chi-squared test for categorical variables. Multiple imputation was applied in addressing missing values for smoking status, alcohol consumption, BMI status, BP status, TC status and Townsend scores. We used multiple imputation by chained equations (MICE) in SAS version 9.4 to create 25 imputed datasets [18]. Rubin’s rules were applied to combine the results from analyses on each of the imputed datasets to produce estimates and confidence intervals [19].

We estimated the risk of mortality presented as hazard ratios (HRs) in relation to the number of medications, medication classes prescribed and different combinations using a marginal structural Cox proportional hazards model, as described by Hernán et al. [20]. This method aims to control for the effects of time-varying confounders and treatment switching. We estimated the parameters of our marginal structural model (MSM) by calculating a weight for each person-year interval and fitting a weighted pooled logistic regression model. Pooled logistic regression approximates the Cox model well when the risk of events is less than 10% per person-time interval [21]; herein, the maximum entry-specific risk of all-cause mortality was only 5.4%.

We used the inverse probability-of-treatment weight and the inverse probability-of-censoring weight to adjust for confounders at each entry point. In weight estimation, the numerator included the time-dependent intercept and the following baseline covariates: sex, baseline age, Townsend score, history of comorbidities and previous cardiovascular medications. The denominator included the time-dependent intercept, the baseline covariates and the following time-varying covariates: age at each entry point, most recently available smoking status, alcohol consumption, BMI status, BP status, TC status, comorbidities and previous occurrence of cardiovascular events (nonfatal MI, angina, stroke or TIA) 1 year prior to each entry point, and time-varying variables of previous cardiovascular medications and NSAIDs use 3 months prior to each entry point.

### Sensitivity analysis

We conducted several sensitivity analyses: (1) using a 60-day screening period instead of a 90-day window, (2) dividing the 1-year follow-up time frame into intervals of 6 months, (3) including patients who had a history of MI before the first stroke or TIA event, (4) repeating the analyses in patients with completed characteristics data (complete-case analyses), (5) categorising missing data for each covariate as a separate group, (6) repeating the analyses separately for patients with TIA and patients with ischemic stroke and (7) an additional sensitivity analysis was conducted to assess the robustness of our findings to unmeasured confounding by computing the *E* value [22]. The *E* value is defined as “the minimum strength of association, on the risk ratio scale, that an unmeasured confounder must have with both the treatment and the outcome to fully explain away a specific treatment-outcome association, conditional on the measured covariates” [22]. All analyses were performed using SAS version 9.4.

## Results

The study cohort consisted of 25,200 men (47.9%) and 27,419 women (52.1%) who experienced an initial ischemic stroke or TIA event from 1 January 2007 to 31 December 2016. Overall, 8.1% of patients did not receive any cardiovascular medications, 9.2% received one, 20.3% received two, 23.0% received three, 19.4% received four, 11.7% received five and 8.2% of patients received six or more cardiovascular medications during the 90 days following their initial ischemic stroke or TIA event. The mean age at the start of follow-up was 72.0 (SD, 11.9) years, and the mean follow-up time was 3.6 (SD, 2.6) years. In total, the study recorded 9230 deaths during follow-up, and the crude death rate was 46.3/1000 person-years. Table 1 shows the baseline characteristics of the patients at their initial ischemic stroke or TIA events based on the number of cardiovascular medications received during the first 90 days. There were significant differences in all characteristics except peptic ulcer disease between the groups.

**Table 1:** Baseline characteristics of study subjects at their initial ischemic stroke or TIA events, 2007–2016

Cardiovascular treatment groups									
	Total, *n* = 52,619	0 medication, *n* = 4259 (8.1%)	1 medication, *n* = 4837 (9.2%)	2 medications, *n* = 10,705 (20.3%)	3 medications, *n* = 12,112 (23.0%)	4 medications, *n* = 10,197 (19.4%)	5 medications, *n* = 6177 (11.7%)	≥ 6 medications, *n* = 4332 (8.2%)	*P* value
**Sex**, % women	27,419 (52.1)	2256 (53.0)	2654 (54.9)	5507 (51.4)	6096 (50.3)	5283 (51.8)	3294 (53.3)	2329 (53.8)	< 0.01
**Age**, (years), mean ± SD	72.0 ± 11.9	71.7 ± 13.3	71.9 ± 13.6	70.7 ± 12.5	71.8 ± 11.7	72.6 ± 11.1	72.9 ± 10.7	73.1 ± 10.6	< 0.01
**Smoking (%)**									
Current	9847 (18.7)	877 (20.6)	911 (18.8)	2252 (21.0)	2401 (19.8)	1822 (17.9)	948 (15.4)	636 (14.7)	
Former	16,458 (31.3)	1186 (27.9)	1349 (27.9)	3144 (29.4)	3855 (31.8)	3304 (32.4)	2121 (34.3)	1499 (34.6)	
Never	24,507 (46.6)	1997 (46.9)	2364 (48.9)	4930 (46.1)	5442 (44.9)	4767 (46.8)	2929 (47.4)	2078 (48.0)	
Missing	1807 (3.4)	199 (4.7)	213 (4.4)	379 (3.5)	414 (3.4)	304 (3.0)	179 (2.9)	119 (2.8)	
**Alcohol (%)**									
Current	26,023 (49.5)	1923 (45.2)	2132 (44.1)	5152 (48.1)	6133 (50.6)	5216 (51.2)	3222 (52.2)	2245 (51.8)	
Former	1728 (3.3)	116 (2.7)	189 (3.9)	355 (3.3)	410 (3.4)	318 (3.1)	213 (3.5)	127 (2.9)	
Never	8658 (16.5)	712 (16.7)	788 (16.3)	1700 (15.9)	1920 (15.9)	1659 (16.3)	1070 (17.3)	809 (18.7)	
Missing	16,210 (30.8)	1508 (35.4)	1728 (35.7)	3498 (32.7)	3649 (30.1)	3004 (29.5)	1672 (27.1)	1151 (26.6)	
**BMI status (%)**									
Normal (18.5–24.9 kg/m^2^)	12,506 (23.8)	1052 (24.7)	1327 (27.4)	2786 (26.0)	2922 (24.1)	2350 (23.1)	1299 (21.0)	770 (17.8)	
Overweight (25.0–29.9 kg/m^2^)	14,897 (28.3)	1080 (25.4)	1229 (25.4)	2879 (26.9)	3408 (28.1)	3062 (30.0)	1933 (31.3)	1306 (30.2)	
Obese (≥ 30.0 kg/m^2^)	11,131 (21.2)	715 (16.8)	670 (13.9)	1748 (16.3)	2410 (19.9)	2382 (23.4)	1727 (28.0)	1479 (34.1)	
Underweight (< 18.5 kg/m^2^)	1075 (2.0)	109 (2.6)	182 (3.8)	268 (2.5)	272 (2.3)	151 (1.5)	58 (0.9)	35 (0.8)	
Missing	13,010 (24.7)	1303 (30.6)	1429 (29.5)	3024 (28.3)	3100 (25.6)	2252 (22.1)	1160 (18.8)	742 (17.1)	
**BP status (%)**									
Normal (BP < 140/90 mmHg)	21,263 (40.4)	1608 (37.8)	2191 (45.3)	4537 (42.4)	4780 (39.5)	3966 (38.9)	2432 (39.4)	1749 (40.4)	
Stage 1 hypertension (BP ≥ 140/90 mmHg)	15,626 (29.7)	1066 (25.0)	1221 (25.2)	2841 (26.5)	3658 (30.2)	3293 (32.3)	2109 (34.1)	1438 (33.2)	
Stage 2 hypertension (BP ≥ 160/100 mmHg)	5166 (9.8)	355 (8.3)	332 (6.9)	766 (7.2)	1198 (9.9)	1184 (11.6)	765 (12.4)	566 (13.1)	
Stage 3 hypertension (systolic BP ≥ 180 mmHg or diastolic BP ≥ 110 mmHg)	2413 (4.6)	154 (3.6)	129 (2.7)	284 (2.7)	508 (4.2)	578 (5.7)	406 (6.6)	354 (8.2)	
Missing	8078 (15.4)	1070 (25.1)	958 (19.8)	2263 (21.1)	1953 (16.1)	1159 (11.4)	454 (7.4)	221 (5.1)	
**TC status (%)**									
Optimal (< 5.2 mmol/L)	16,562 (31.5)	995 (23.4)	1092 (22.6)	2648 (24.7)	3560 (29.4)	3636 (35.7)	2571 (41.6)	2060 (47.6)	
Intermediate (5.3–6.2 mmol/L)	7898 (15.0)	519 (12.2)	626 (12.9)	1596 (14.9)	1929 (15.9)	1598 (15.7)	974 (15.8)	656 (15.1)	
High (> 6.2 mmol/L)	4510 (8.6)	314 (7.4)	386 (8.0)	921 (8.6)	1111 (9.2)	902 (8.9)	551 (8.9)	325 (7.5)	
Missing	23,649 (44.9)	2431 (57.1)	2733 (56.5)	5540 (51.8)	5512 (45.5)	4061 (39.8)	2081 (33.7)	1291 (29.8)	
**Townsend score (%)**									
1 (least deprived)	10,959 (20.8)	809 (19.0)	1037 (21.4)	2256 (21.1)	2627 (21.7)	2155 (21.1)	1248 (20.2)	827 (19.1)	
2	10,833 (20.6)	851 (20.0)	1058 (21.9)	2216 (20.7)	2496 (20.6)	2083 (20.4)	1306 (21.1)	823 (19.0)	
3	9949 (18.9)	827 (19.4)	952 (19.7)	2051 (19.2)	2168 (17.9)	1932 (19.0)	1173 (19.0)	846 (19.5)	
4	8613 (16.4)	745 (17.5)	734 (15.2)	1716 (16.0)	2011 (16.6)	1639 (16.1)	1004 (16.3)	764 (17.6)	
5 (most deprived)	5995 (11.4)	494 (11.6)	515 (10.7)	1255 (11.7)	1400 (11.6)	1113 (10.9)	724 (11.7)	494 (11.4)	
Missing	6270 (11.9)	533 (12.5)	541 (11.2)	1211 (11.3)	1410 (11.6)	1275 (12.5)	722 (11.7)	578 (13.3)	
**History of PCI (%)**	262 (0.5)	13 (0.3)	6 (0.1)	26 (0.2)	42 (0.4)	51 (0.5)	43 (0.7)	81 (1.9)	
**Comorbidity (%)**									
Hypertension	29,382 (55.8)	1802 (42.3)	1604 (33.2)	3547 (33.1)	6353 (52.5)	7208 (70.7)	5058 (81.9)	3810 (88.0)	< 0.01
Hyperlipidaemia	7510 (14.3)	433 (10.2)	463 (9.6)	1257 (11.7)	1644 (13.6)	1629 (16.0)	1187 (19.2)	897 (20.7)	< 0.01
Arrhythmia	8159 (15.5)	645 (15.1)	449 (9.3)	1095 (10.2)	1611 (13.3)	1851 (18.2)	1331 (21.6)	1177 (27.2)	< 0.01
Heart failure	2235 (4.3)	154 (3.6)	98 (2.0)	233 (2.2)	373 (3.1)	446 (4.4)	415 (6.7)	516 (11.9)	< 0.01
PVD	2752 (5.2)	209 (4.9)	178 (3.7)	450 (4.2)	557 (4.6)	583 (5.7)	427 (6.9)	348 (8.0)	< 0.01
Diabetes	8921 (17.0)	568 (13.3)	511 (10.6)	1313 (12.3)	1845 (15.2)	1933 (19.0)	1442 (23.3)	1309 (30.2)	< 0.01
Dementia	2549 (4.8)	271 (6.4)	518 (10.7)	653 (6.1)	518 (4.3)	324 (3.2)	170 (2.8)	95 (2.2)	< 0.01
COPD	4424 (8.4)	297 (7.0)	412 (8.5)	881 (8.2)	1058 (8.7)	892 (8.8)	517 (8.4)	367 (8.5)	0.02
Asthma	6888 (13.1)	494 (11.6)	679 (14.0)	1418 (13.3)	1642 (13.6)	1292 (12.7)	789 (12.8)	574 (13.3)	< 0.01
Liver disease	338 (0.6)	45 (1.1)	45 (0.9)	59 (0.6)	79 (0.7)	61 (0.6)	29 (0.5)	20 (0.5)	< 0.01
Peptic ulcer disease	2974 (5.7)	240 (5.6)	282 (5.8)	594 (5.6)	689 (5.7)	587 (5.8)	346 (5.6)	236 (5.5)	0.98
RA	1094 (2.1)	96 (2.3)	113 (2.3)	199 (1.9)	259 (2.1)	206 (2.0)	132 (2.1)	89 (2.1)	0.51
CKD	9366 (17.8)	666 (15.6)	597 (12.4)	1346 (12.7)	1922 (16.0)	2045 (20.1)	1495 (24.2)	1259 (29.2)	< 0.01

*BMI* body mass index, *BP* blood pressure, *TC* total cholesterol, *COPD* chronic obstructive pulmonary disease, *CKD* chronic kidney disease, *PCI* percutaneous transluminal coronary intervention, *PVD* peripheral vascular disease, *RA* rheumatoid arthritis

**Fig. 1:**
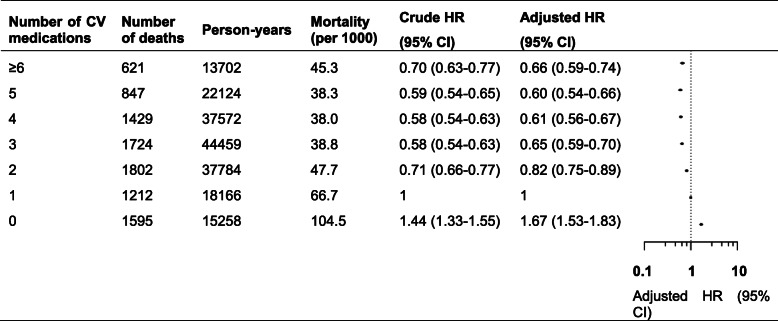
Risk of all-cause mortality in patients prescribed cardiovascular medications. Mortality indicates unadjusted absolute risk per 1000 person-years. Crude HR was assessed by an unweighted pooled logistic regression without any adjustment for confounding. Adjusted HR was assessed by the MSMs adjusted for time-invariant and time-varying confounders

**Fig. 2:**
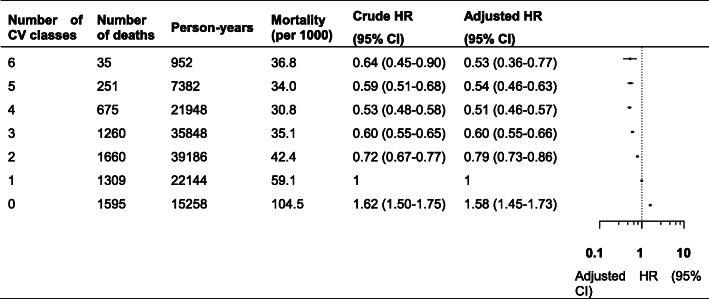
Risk of all-cause mortality in patients prescribed six specific classes of cardiovascular medications. Mortality indicates unadjusted absolute risk per 1000 person-years. Crude HR was assessed by an unweighted pooled logistic regression without any adjustment for confounding. Adjusted HR was assessed by the MSMs adjusted for time-invariant and time-varying confounders

Figure 1 shows the risk of all-cause mortality in patients prescribed with different numbers of cardiovascular medications. Compared with monotherapy, the risk of all-cause mortality was lower in patients with combination therapy: 18% (95% CI 11–25%) lower with two medications, 35% (95% CI 30–41%) lower with three medications, 39% (95% CI 33–44%) lower with four medications, 40% (95% CI 34–46%) lower with five medications and 34% (95% CI 26–41%) lower with six or more medications. Conversely, no use of cardiovascular medications was associated with an increased risk of all-cause mortality (adjusted HR 1.67, 95% CI 1.53–1.82). Similar results were found for the different numbers of cardiovascular medication classes. Figure 2 shows the decreased risks of mortality in patients with two (adjusted HR 0.79, 95% CI 0.73–0.86), three (adjusted HR 0.60, 95% CI 0.55–0.66), four (adjusted HR 0.51, 95% CI 0.46–0.57), five (adjusted HR 0.54, 95% CI 0.46–0.63) and six (adjusted HR 0.53, 95% CI 0.36–0.77) specific classes of cardiovascular medications compared with patients prescribed one class. Patients with a four-class combination had the lowest risk of mortality.

**Fig. 3:**
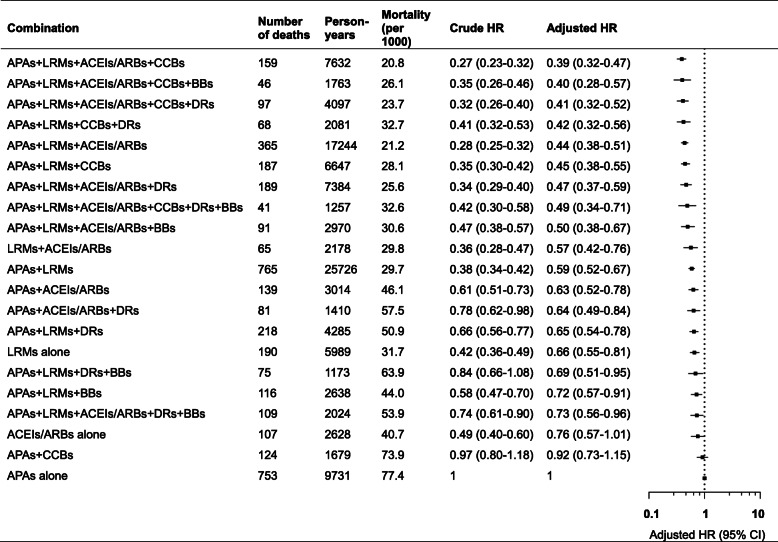
Risk of all-cause mortality in the 20 most commonly used regimens containing the six specific classes of cardiovascular medications compared with antiplatelet agents alone. Mortality indicates unadjusted absolute risk per 1000 person-years. Crude HR was assessed by an unweighted pooled logistic regression without any adjustment for confounding. Adjusted HR was assessed by the MSMs adjusted for time-invariant and time-varying confounders

In the analysis of the effect of the 20 most commonly used regimens containing APAs, LRMs, ACEIs/ARBs, CCBs, DRs and BBs versus APAs alone, we found a significantly lower risk of mortality in combinations containing APAs, LRMs, ACEIs/ARBs and CCBs (Fig. 3). In patients with the combination treatment of APAs, LRMs, ACEIs/ARBs and CCBs, the risk of mortality was lowered by 61% (95% CI 53–68%) compared with APAs alone. When adding BBs or DRs to this four-medication combination, the risk of mortality was lowered by 60% (95% CI 43–72%) and 59% (95% CI 48–68%)**,** respectively**,** when compared to APAs alone. The combination of only three classes of APAs, LRMs and ACEIs/ARBs also showed a significantly lower risk of mortality with an adjusted HR of 0.44 (95% CI 0.38–0.51).

### Sensitivity analyses

The results of sensitivity analyses are provided in Additional file 1: Table S1-S2. Our primary results of the risk of mortality in patients with different numbers of cardiovascular medications and different numbers of classes of cardiovascular medications are similar to the results in the analysis using a 60-day exposure window. The analyses in patients with a history of MI, patients with competing risk characteristic data, when categorising missing data as a separate group and in separate analyses among patients with TIA only and patients with ischemic stroke only were consistent with the results of primary analyses. The results showed an even lower risk of mortality in patients with combination therapy when the follow-up duration was divided into 6-month intervals. The *E* values (risk ratios) for the three main analyses of all-cause mortality ranged from 1.74 to 4.57.

## Discussion

This cohort study is the first large, long follow-up database-based study to report the effectiveness of increasing numbers, classes and combinations of cardiovascular medications in the secondary prevention of all-cause mortality in patients who experienced an incident ischemic stroke or TIA. Our results showed that increasing the numbers and classes of cardiovascular medications appeared to produce additional benefits on long-term survival. APAs, LRMs, ACEIs/ARBs and CCBs appeared to be the optimal constituents of combination therapy associated with reduced risk of mortality after stroke or TIA.

Previous studies have suggested the benefit of the management of single risk factors such as hypertension, high cholesterol and thrombus formation in the secondary prevention of stroke [6, 23, 24]. Our results strongly suggest that multiple pharmacological interventions can provide potentially greater benefits on long-term survival for stroke patients. The results showed that HR of mortality reached a plateau in patients with four (0.61, 95% CI 0.56–0.67) or five (0.60, 95% CI 0.54–0.66) medications. Contrary to the combination therapy, patients with no use of cardiovascular medications had a higher risk of mortality. In summary, the combined use of four or five cardiovascular medications in the present study appeared optimal to improve long-term survival after stroke.

Evidence-based guidelines recommend APAs, LRMs and antihypertension medications for the secondary prevention of stroke and TIA [25]. Diuretics, ACEIs/ARBs and CCBs are first-line antihypertensive medications [26]. Our study identified the priority of APAs, LRMs, ACEIs/ARBs and CCBs in the secondary prevention of stroke, which is consistent with the current guideline recommendations. This four-medication combination was associated with a 61% reduction in mortality compared with APAs alone. The 2-year retrospective cohort study of Park and Ovbiagele [27] suggested that the combination of antihypertensive medications, anti-thrombotic medications and lipid modifiers was associated with a significant reduction of death following an occurrence of stroke. The study classified several classes of cardiovascular medications such as ACEIs/ARBs, CCBs, DRs and BBs as antihypertensive medications. However, our study did not find a significant additional benefit when beta-blockers were added to the combination therapy on long-term survival. This is in line with a systematic review of RCTs [28], in which no clear evidence supported a beneficial effect of beta-blockers for secondary prevention of stroke or TIA.

In addition, our results highlighted an issue that the use of cardiovascular medications for the secondary prevention of stroke and TIA remained sub-optimal. In our study, 8.1% of patients did not receive long-term use of cardiovascular medications, and 9.2% received only monotherapy following their first stroke or TIA event. Other studies in the UK population have also indicated the underuse of evidence-based pharmacotherapy for cardiovascular disease [29, 30]. We investigated demographics and clinical characteristics at each entry point during the follow-up period. Patients with no or one cardiovascular medication were mostly at a relatively lower risk of cardiovascular disease (e.g. younger age, normal BMI status, with fewer comorbidities) compared with patients with three or more drugs (Additional file 1: Table S3). However, we could not rule out the missing data issue here as aspirin is widely available over-the-counter, and there may be some patients who had been admitted to hospitals; therefore, the cardiovascular medication during that period would not be available in the GP record. Previous studies also have demonstrated that cardiovascular risk levels [8], concerns on treatment risk (e.g. side effects) [31] and patients preferences [32, 33] may explain the discrepancy between the guidelines and real-world clinical practice. Our results have strengthened the evidence for the long-term beneficial effects of combined guideline-recommended cardiovascular medications. We demonstrated that pharmacotherapy in secondary prevention is necessary and beneficial for individuals who have had a stroke regardless of the risk level of cardiovascular disease. We suggest that guideline compliance deserves better attention to improve survival in patients with stroke or TIA.

### Strengths and limitations

This study has several strengths. Firstly, this study was based on a large population-based primary care practice database. As such, it is likely to reflect the usual healthcare in the UK. Secondly, this study compared different numbers, classes and combinations of cardiovascular medications which comprehensively demonstrated the effect of combination therapy on long-term survival. Thirdly, when assessing the effect of different combinations, we defined exposure groups as patients who were exclusively using the selected cardiovascular medications of interest, and this was to remove potential effects of other cardiovascular medications which were not of interest on the outcome. In addition, we used MSMs to control for confounding due to both time-invariant and time-varying confounders that may lead to treatment switching or informative censoring. We demonstrated the robustness of our findings to unmeasured confounding using the *E* value estimate. Most HRs of all-cause mortality for known, strong risk factors of cardiovascular disease were below 1.74, the minimum *E* value in this study. For example, the HRs of mortality were 1.61 (95% CI 1.49–1.74) for current smokers, 1.27 (95% CI 1.19–1.36) for patients with diabetes and 1.14 (95% CI 1.07–1.20) for patients with hypertension. It is not likely that an unmeasured or unknown confounder would have a substantially larger effect on cardiovascular disease development or mortality than these known risk factors by having a relative risk exceeding 1.74. Finally, most compellingly, we used all-cause mortality as our outcome measure. Despite the influence of noncardiovascular mortality on the outcome, our study produced very clear results. Had we measured cause-specific cardiovascular mortality, we suspect that our findings would have been more pronounced.

This study has limitations. Firstly, the THIN database only provides records of prescriptions; therefore, our study was not able to determine if medications were actually dispensed, taken or used in line with the administration directions by patients. Secondly, because the THIN database does not capture data for hospital treatment, treatment in some care homes or nursing homes, and over the counter (OTC) medications (e.g. aspirin available OTC), the study was not able to address any medication usage not included in records from general practice. Thirdly, we had no information on the severity of stroke. Due to shorter life-expectancy, health interventions may be less cost-effective in patients with more severe cardiovascular conditions [34, 35]. In this case, patients with severe stroke may be more likely to be undertreated and thus more likely to die. However, we adopted measures to balance the heterogeneity between different exposure groups to some extent: (1) we excluded patients who had a history of MI before the first stroke event, (2) excluded patients who died or had a nonfatal cardiovascular event during the first 90 days after stroke or TIA and (3) we adjusted for risk factors of cardiovascular disease when estimating mortality hazard ratios. Fourthly, we only estimated the effect of cardiovascular medications by their major classification so our study cannot tell the effect of sub-classes of these cardiovascular medications on long-term outcomes. For instance, previous systematic reviews have suggested that dual antiplatelet therapy was more effective on short-term outcomes than monotherapy in stroke patients [9–11], but our study did not compare the effect of dual-antiplatelet therapy and monotherapy on long-term mortality. Further research is required to explore this area. In addition, the clinical guidelines of pharmacotherapy for secondary prevention of stroke had no major changes over the period of 2007–2016 (refer to guidelines from AHA/ASA 2006 [36], 2010 [37] and 2014 [4]; National clinical guideline for stroke 2008 [38], 2012 [39] and 2016 [40]). There are some changes of recommendations on dosage and individual drugs. For example, in terms of lipid-lowering therapy in secondary prevention, the National Clinical Guideline 2008 recommended using statins according to a recommended cholesterol level. Guideline 2012 recommended high-intensity statin use such as atorvastatin 20–80 mg daily and Guideline 2016 recommended initiated using a statin with low acquisition cost such as simvastatin 40 mg daily. Our study only focused on the numbers and classes of CV drugs and did not address the dosage issue in the study due to the complexity of the research question and analysis. There may be some residual confounding impact on the mortality outcome in our study. But we would expect this impact is minimal. Future studies on drug dosage are encouraged.

## Conclusions

Our study suggests that combination therapy of four or five cardiovascular medications may be optimal for long-term survival in patients with stroke or TIA. APAs, LRMs, ACEIs/ARBs and CCBs were the optimal constituents of combination therapy in the present study.

## Supplementary Information

**Additional file 1: Table S1.** Results for various numbers of cardiovascular medications in sensitivity analyses. **Table S2.** Results for various numbers of specific medication classes in sensitivity analyses. **Table S3.** Summary of characteristics of study subjects with competed data.

## Data Availability

THIN data is not available to the public.
