# Correction to: Impact of multiple cardiovascular medications on mortality after an incidence of ischemic stroke or transient ischemic attack

**DOI:** 10.1186/s12916-021-02113-2

**Published:** 2021-09-12

**Authors:** Tian-Tian Ma, Ian C. K. Wong, Cate Whittlesea, Kenneth K. C. Man, Wallis Lau, Zixuan Wang, Ruth Brauer, Thomas M. MacDonald, Isla S. Mackenzie, Li Wei

**Affiliations:** 1 Research Department of Practice and Policy, School of Pharmacy, University, College London, London, UK; 2 Centre for Safe Medication Practice and Research, Department of Pharmacology and Pharmacy, Li Ka Shing Faculty of Medicine, The University of Hong Kong, Pok Fu Lam, Hong Kong; 3 Medicines Monitoring Unit (MEMO Research) and Hypertension Research Centre, University of Dundee, Dundee, UK

**Correction to: BMC Med 19, 24 (2021)**

**https://doi.org/10.1186/s12916-021-01900-1**

The authors of the original article [1] wish to amend the text in the **Study design** sub-section of the **Methods** section.

The text in this section should instead state the following:

“A cohort study was conducted using electronic primary healthcare data from the IQVIA Medical Research Data (IMRD) that incorporates data supplied by THIN (The Health Improvement Network), a propriety database of Cegedim SA.”
