# Supplementary material for: Impact of multiple cardiovascular medications on mortality after an incidence of ischemic stroke or transient ischemic attack
Source: BMC Med. 2021 Feb 3;19:24. doi: 10.1186/s12916-021-01900-1 (PMC7856718; doi:10.1186/s12916-021-01900-1)
Supplement: Supplementary file 1 — Additional file 1: Table S1. Results for various numbers of cardiovascular medications in sensitivity analyses. Table S2. Results for various numbers of specific medication classes in sensitivity analyses. Table S3. Summary of characteristics of study subjects with competed data. [file 12916_2021_1900_MOESM1_ESM.docx]

## Electronic supplementary material

**Impact of multiple cardiovascular medications on mortality after an incidence of ischemic stroke or TIA**

Tian-Tian Ma^1^; Ian CK Wong^1,2^; Cate Whittlesea^1^; Kenneth KC Man^1,2^; Wallis Lau^1,2^; Zixuan Wang^1^; Ruth Brauer^1^; Thomas M MacDonald^3^; Isla S Mackenzie^3^; Li Wei^1*^.

**Authors’ affiliations:**

^1^Research Department of Practice and Policy, UCL School of Pharmacy, London, United Kingdom;

^2^Centre for Safe Medication Practice and Research, Department of Pharmacology and Pharmacy, Li Ka Shing Faculty of Medicine, The University of Hong Kong, Hong Kong;

^3^Medicines Monitoring Unit (MEMO Research) and Hypertension Research Centre, University of Dundee, Dundee, United Kingdom.

***Corresponding author:**

Professor Li Wei,

UCL School of Pharmacy, 29-39 Brunswick Square, London, WC1N 1AX, United Kingdom

Email: [l.wei@ucl.ac.uk](mailto:l.wei@ucl.ac.uk)

**Table S1. Risk of all-cause mortality in patients prescribed with various numbers of cardiovascular medications in sensitivity analyses**

| **Adjusted HR (95% CI)** | | | | | | | | |
| --- | --- | --- | --- | --- | --- | --- | --- | --- |
| **Number of CV medications** | **Primary analysis** | **60-day exposure window^a^** | **6-month interval**^b^ | **With history of MI^c^** | **Complete data^d^** | **Categorised missing data^e^** | **Patients with TIA^f^** | **Patients with stroke^g^** |
| ≥6 | 0.66 (0.59-0.74) | 0.73 (0.64-0.82) | 0.48 (0.42-0.54) | 0.63 (0.56-0.71) | 0.64 (0.52-0.79) | 0.68 (0.61-0.76) | 0.65 (0.54-0.79) | 0.64 (0.56-0.75) |
| 5 | 0.60 (0.54-0.66) | 0.62 (0.56-0.70) | 0.46 (0.41-0.51) | 0.57 (0.51-0.64) | 0.64 (0.52-0.78) | 0.60 (0.54-0.66) | 0.54 (0.46-0.64) | 0.58 (0.51-1.67) |
| 4 | 0.61 (0.56-0.67) | 0.63 (0.58-0.68) | 0.48 (0.44-0.53) | 0.59 (0.53-0.64) | 0.59 (0.49-0.71) | 0.62 (0.57-0.68) | 0.56 (0.48-0.64) | 0.63 (0.56-0.71) |
| 3 | 0.65 (0.59-0.70) | 0.66 (0.60-0.72) | 0.53 (0.48-0.57) | 0.62 (0.56-0.68) | 0.62 (0.52-0.74) | 0.65 (0.60-0.70) | 0.64 (0.56-0.73) | 0.63 (0.56-0.70) |
| 2 | 0.82 (0.75-0.89) | 0.82 (0.75-0.89) | 0.69 (0.64-0.75) | 0.78 (0.71-0.85) | 0.82 (0.69-0.98) | 0.82 (0.76-0.89) | 0.78 (0.69-0.89) | 0.84 (0.75-0.93) |
| 1 | 1 | 1 | 1 | 1 | 1 | 1 | 1 | 1 |
| 0 | 1.67 (1.53-1.83) | 1.60 (1.45-1.76) | 2.16 (1.99-2.34) | 1.65 (1.50-1.81) | 1.88 (1.56-2.26) | 1.58 (1.45-1.73) | 1.58 (1.37-1.82) | 1.64 (1.46-1.84) |

^a^A sensitivity analysis conducted in a 60-day exposure period.

^b^A sensitivity analysis conducted in 6-month intervals.

^c^A sensitivity analysis conducted in patients who had a history of MI before the first stroke or TIA event.

^d^A sensitivity analysis conducted in patients with complete characteristics data.

^e^A sensitivity analysis conducted by categorising missing data as a separate group.

^f^A sensitivity analysis conducted in patients with the first TIA event

^g^A sensitivity analysis conducted in patients with the first ischemic stroke event

**Table S2. Risk of all-cause mortality in patients prescribed with various numbers of specific six classes of cardiovascular medications in sensitivity analyses**

| **Adjusted HR (95% CI)** | | | | | | | | |
| --- | --- | --- | --- | --- | --- | --- | --- | --- |
| **Number of classes** | **Primary analysis** | **60-day exposure window^a^** | **6-month interval**^b^ | **With history of MI^c^** | **Complete data^d^** | **Categorised missing data^e^** | **Patients with TIA^f^** | **Patients with stroke^g^** |
| 6 | 0.53 (0.36-0.77) | 0.66 (0.59-0.74) | 0.47 (0.27-0.81) | 0.61 (0.38-0.97) | 0.58 (0.31-1.08) | 0.73 (0.48-1.09) | 0.82 (0.41-1.63) | 1.07 (0.55-2.08) |
| 5 | 0.54 (0.46-0.63) | 0.60 (0.54-0.66) | 0.40 (0.33-0.47) | 0.56 (0.47-0.65) | 0.67 (0.51-0.86) | 0.73 (0.61-0.86) | 0.62 (0.46-0.83) | 0.77 (0.57-1.04) |
| 4 | 0.51 (0.46-0.57) | 0.61 (0.56-0.67) | 0.42 (0.38-0.47) | 0.51 (0.45-0.58) | 0.59 (0.49-0.72) | 0.63 (0.56-0.71) | 0.52 (0.44-0.63) | 0.64 (0.53-0.78) |
| 3 | 0.60 (0.55-0.66) | 0.65 (0.59-0.70) | 0.50 (0.45-0.54) | 0.58 (0.52-0.65) | 0.62 (0.53-0.74) | 0.69 (0.62-0.76) | 0.62 (0.53-0.72) | 0.67 (0.58-0.78) |
| 2 | 0.79 (0.73-0.86) | 0.82 (0.75-0.89) | 0.67 (0.61-0.72) | 0.76 (0.69-0.84) | 0.80 (0.68-0.94) | 0.76 (0.68-0.85) | 0.77 (0.68-0.88) | 0.86 (0.75-0.98) |
| 1 | 1 | 1 | 1 | 1 | 1 | 1 | 1 | 1 |
| 0 | 1.58 (1.45-1.73) | 1.67 (1.53-1.83) | 2.12 (1.96-2.29) | 1.59 (1.41-1.79) | 1.97 (0.66-2.34) | 1.17 (0.92-1.47) | 1.58 (0.31-1.92) | 1.56 (1.34-1.81) |

^a^A sensitivity analysis conducted in a 60-day exposure period.

^b^A sensitivity analysis conducted in 6-month intervals.

^c^A sensitivity analysis conducted in patients who had a history of MI before the first stroke or TIA event.

^d^A sensitivity analysis conducted in patients with complete characteristics data.

^e^A sensitivity analysis conducted by categorising missing data as a separate group.

^f^A sensitivity analysis conducted in patients with the first TIA event

^g^A sensitivity analysis conducted in patients with the first ischemic stroke event

**Table S3. Summary of characteristics of study subjects with competed data at all the entry points**

| **Cardiovascular treatment groups** | | | | | | | | |
| --- | --- | --- | --- | --- | --- | --- | --- | --- |
|  | **0 drug** | **1 drug** | **2 drugs** | **3 drugs** | **4 drugs** | **5 drugs** | **≥6 drugs** | **P value** |
| **Sex**, % women | 49.2 | 49.8 | 47.8 | 47.3 | 48.0 | 48.6 | 45.8 | <0.01 |
| **Age**, (years) mean ± SD | 69.7 ± 12.1 | 70.7 ± 11.7 | 70.8± 11.0 | 71.7 ± 10.5 | 72.3 ± 10.1 | 72.2 ± 10.1 | 71.9 ± 9.9 | <0.01 |
| **Smoking (%)** |  |  |  |  |  |  |  |  |
| Current | 18.5 | 16.2 | 17.3 | 16.0 | 14.9 | 12.7 | 12.5 | <0.01 |
| Former | 40.1 | 39.8 | 40.1 | 42.4 | 44.0 | 44.5 | 45.8 |  |
| Never | 41.4 | 43.9 | 42.6 | 41.6 | 41.1 | 42.8 | 41.7 |  |
| **Alcohol (%)** |  |  |  |  |  |  |  |  |
| Current | 70.0 | 68.9 | 70.4 | 71.4 | 70.9 | 69.2 | 70.4 | <0.01 |
| Former | 11.1 | 12.1 | 12.1 | 11.2 | 12.0 | 11.4 | 10.6 |  |
| Never | 18.9 | 19.0 | 17.5 | 17.4 | 17.1 | 19.4 | 19.1 |  |
| **BMI status (%)** |  |  |  |  |  |  |  |  |
| Normal | 35.6 | 35.8 | 33.3 | 30.1 | 26.6 | 23.1 | 19.1 | <0.01 |
| Overweight | 36.9 | 37.9 | 39.2 | 39.7 | 38.7 | 37.9 | 35.7 |  |
| Obesity | 23.9 | 23.3 | 25.2 | 28.4 | 33.1 | 37.6 | 44.2 |  |
| Underweight | 3.6 | 3.0 | 2.4 | 1.8 | 1.6 | 1.4 | 1.0 |  |
| **BP status (%)** |  |  |  |  |  |  |  |  |
| Normal | 64.8 | 66.4 | 66.5 | 63.2 | 60.9 | 59.5 | 57.7 | <0.01 |
| Stage 1 hypertension | 27.5 | 27.4 | 27.8 | 29.8 | 30.7 | 30.5 | 30.5 |  |
| Stage 2 hypertension | 5.5 | 4.9 | 4.3 | 5.4 | 6.3 | 7.4 | 8.3 |  |
| Stage 3 hypertension | 2.0 | 1.3 | 1.3 | 1.6 | 2.1 | 2.5 | 3.4 |  |
| **TC status (%)** |  |  |  |  |  |  |  |  |
| Optimal | 62.6 | 64.8 | 73.4 | 77.5 | 80.6 | 81.8 | 82.7 | <0.01 |
| Intermediate | 25.1 | 23.9 | 18.1 | 15.8 | 13.7 | 12.7 | 12.1 |  |
| High | 12.2 | 11.3 | 8.5 | 6.7 | 5.7 | 5.5 | 5.2 |  |
| **Townsend score (%)** |  |  |  |  |  |  |  |  |
| 1 (least deprived) | 23.0 | 22.0 | 22.5 | 22.0 | 21.2 | 19.9 | 20.3 | <0.01 |
| 2 | 22.2 | 24.3 | 22.6 | 22.6 | 22.9 | 23.6 | 22.3 |  |
| 3 | 22.4 | 22.3 | 21.9 | 21.8 | 21.9 | 21.0 | 22.1 |  |
| 4 | 18.0 | 18.9 | 19.0 | 19.3 | 19.8 | 19.9 | 20.7 |  |
| 5 (most deprived) | 14.3 | 12.6 | 14.0 | 14.4 | 14.3 | 15.6 | 14.6 |  |
| **History of PCI (%)** | 0.4 | 0.3 | 0.3 | 0.5 | 1.0 | 1.9 | 3.0 |  |
| **Comorbidity (%)** |  |  |  |  |  |  |  |  |
| Hypertension | 48.4 | 43.7 | 46.8 | 67.9 | 81.4 | 88.4 | 91.8 | <0.01 |
| Hyperlipidaemia | 15.5 | 16.2 | 17.7 | 18.8 | 20.6 | 23.1 | 25.8 | <0.01 |
| Arrhythmia | 12.6 | 11.2 | 11.6 | 15.3 | 20.7 | 24.4 | 30.4 | <0.01 |
| Heart Failure | 3.4 | 2.5 | 2.4 | 3.7 | 5.8 | 9.0 | 15.2 | <0.01 |
| PVD | 6.0 | 4.2 | 5.5 | 6.3 | 7.1 | 8.3 | 10.2 | <0.01 |
| Diabetes | 22.2 | 21.0 | 23.1 | 27.3 | 30.5 | 37.1 | 46.9 | <0.01 |
| Dementia | 5.9 | 5.7 | 5.5 | 4.1 | 3.4 | 3.3 | 2.6 | <0.01 |
| COPD | 9.6 | 11.5 | 11.7 | 10.9 | 11.7 | 9.7 | 10.5 | <0.01 |
| Asthma | 12.9 | 15.6 | 16.1 | 15.2 | 14.5 | 13.9 | 12.8 | <0.01 |
| Liver disease | 1.4 | 1.1 | 0.8 | 0.8 | 0.6 | 0.5 | 0.5 | <0.01 |
| Peptic ulcer disease | 6.6 | 5.9 | 6.5 | 6.8 | 6.2 | 5.9 | 6.3 | 0.09 |
| RA | 2.4 | 2.4 | 2.1 | 2.4 | 2.2 | 1.7 | 2.1 | 0.03 |
| CKD | 17.4 | 17.0 | 17.8 | 20.8 | 25.6 | 30.2 | 35.2 | <0.01 |

Abbreviations: BMI, body mass index; BP, blood pressure; TC, total cholesterol; COPD, chronic obstructive pulmonary disease; CKD, chronic kidney disease; PCI, percutaneous transluminal coronary intervention; PVD, peripheral vascular disease; RA, rheumatoid arthritis
